# Implementation suggestions for shared decision-making: results from a comparative study of inpatients and outpatients experience surveys

**DOI:** 10.1186/s12913-025-12507-0

**Published:** 2025-03-11

**Authors:** Cindy Yue Tian, Eliza Lai-Yi Wong, Hong Qiu, Shimeng Liu, Kailu Wang, Yan Wei, Annie Wai-Ling Cheung, Yingyao Chen, Eng-Kiong Yeoh

**Affiliations:** 1 JC School of Public Health and Primary Care, Faculty of Medicine, The Chinese University of Hong Kong, Hong Kong, Hong Kong Special Administrative Region China; 2 Centre for Health Systems and Policy Research, JC School of Public Health and Primary Care, The Chinese University of Hong Kong, Hong Kong, Hong Kong Special Administrative Region China; 3 School of Public Health, Fudan University, Shanghai, 200032 China; 4 National Health Commission Key Laboratory of Health Technology Assessment, Fudan University, Shanghai, 200032 China

**Keywords:** Shared decision-making, General practice, Patient experience, Doctor’s care quality, Trust in doctors

## Abstract

**Background:**

Shared decision-making (SDM) is crucial in patient-centered healthcare services, but its integration into routine medical care remains limited. This study aimed to investigate patients’ experience with SDM in both outpatient and inpatient settings, exploring how the quality of care provided by doctors and patient’s trust in doctors influence SDM across different contexts.

**Methods:**

This study utilized data from the regional cross-sectional surveys, including the 2019 Inpatient Experience and the 2021 Specialist Outpatient Experience survey in Hong Kong. Multivariable logistic regression and path analysis were conducted.

**Results:**

A total of 20,675 participants were included (inpatients: *n* = 8,275; outpatients: *n* = 12, 400) in this study. The results indicated that inpatients perceiving better quality of doctor’s care were significantly more likely to participate in SDM (OR = 1.29, 95%CI = 1.26–1.47, *p* < 0.001), with trust in doctors significantly moderating this association. Conversely, among outpatients, a higher quality of doctor’s care was significantly associated with decreased SDM involvement (OR = 0.91, 95% CI = 0.88-1.00, *p* = 0.04), with trust in doctors serving as a mediator in suppressing this association. Additionally, both subsets indicated that females, the elderly, individuals with good health status, less-educated people, and those living alone were less likely to engage in SDM.

**Conclusion:**

These findings underscore the importance of tailoring SDM implementation to specific contexts, acknowledging the different challenges within outpatient and inpatient settings. Building trust is key to promoting SDM, with added support for vulnerable groups to ensure their involvement in decision-making.

**Supplementary Information:**

The online version contains supplementary material available at 10.1186/s12913-025-12507-0.

## Background

For over three decades, shared decision-making (SDM) has been widely aknowledged to enhance the daily implementation of informed consent in medical treatments and patient-centered healthcare services [1]. The process of SDM involves a mutual exchange of information, preferences, and concerns between healthcare providers and patients [2], with the final goal being to reach a decision that aligns with the patient’s values and preferences while being grounded in clinical evidence [2]. Through such a process, SDM can lead to better treatment compliance, higher satisfaction with healthcare, and improved health outcomes [3–5]. However, the implementation of SDM in routine medical care remains problematic, as previous empirical research in diverse patient groups has revealed limited levels of patient participation in decision-making [6–13]. For example, Nuwagaba et al. reported that only 11.3% of outpatient participants exhibited satisfactory engagement in SDM [9]. One cross-sectional survey involving 1,000 participants reported that 41% of respondents perceived that their doctor made the final decision, instead of engaging in SDM [7].

Research on SDM has investigated various factors influencing its implementation, focusing on patient-level variables (e.g., age [14, 15], gender [16, 17], education [18]) and physician-level factors (e.g., gender [19] and training experience [20]). However, context-level factors have received less attention in existing studies. Understanding the role of context is critical in implementation science and health services improvement [21–25]. The Normalization Process Theory (NPT) points out that whether new habits or practices become a regular part of daily life, heavily depends on the specific details of the contexts in which they are introduced [26]. In simpler terms, the theory suggests that the success or failure of making something a routine is not just about the practice itself but also about how well it fits into the surrounding social and organizational setting. Therefore, it is possible that the implementation of SDM, which might be straightforward in certain places, could face challenges in others. Building upon this theory and further taking into account the findings of earlier studies, which highlighted that factors such as the severity of patient diseases [15, 16], types of decision-making [27], and duration of medical consultation [10, 15] may contribute to the complexity of decision-making processes, outpatient and inpatient settings represent different challenges and opportunities for the effective implementation of SDM.

NPT also emphasizes the interaction between those adopting or rejecting the practice (subjects/patients) and those driving the change and trying to normalize the practice (agents/doctors), as this interaction determines how well the practice is understood, accepted, and sustained in routine care [26]. With this in mind, successful implementation of SDM depends on how well these doctors can engage with patients. Regarding this interaction, this study focused on two key factors: doctor’s care quality and patient’s trust in doctors. Doctor’s care quality has been recognised to be an important factor affecting SDM [28, 29]. This being the case, some studies suggest that high-quality care may not engage patients in decision-making if patients prefer to delegate decisions to their doctors [30]. Additionally, some empirical studies have suggested that physician communication skills had no significant association with patient engagement in decision-making [31, 32]. Therefore, conclusions foregrounding inclusivity in the association between doctor’s care quality and patient engagement in SDM cannot be drawn, highlighting a need for further investigation.

Patient’s trust in doctors (trust in doctors) can be understood either as a reflection of the medical profession as a whole or in the context of the specific interpersonal relationship between an individual doctor and a patient [33]. Importantly, interpersonal patient trust in doctors has been found to have an impact on patient engagement in SDM. For instance, one study involving 2,197 inpatients reported that increased trust in clinicians has been significantly associated with a greater degree of patient-reported SDM [34]. However, other studies failed to find a significant correlation between significant association between trust in doctors and either patient-reported or observer-rated SDM [35]. Additionally, some researchers suggested that “blind trust” may be linked to patient preferences for a passive role in decision-making [36]. These inconsistent findings highlighted the complexity of the relationship between trust and SDM.

**Fig. 1:**
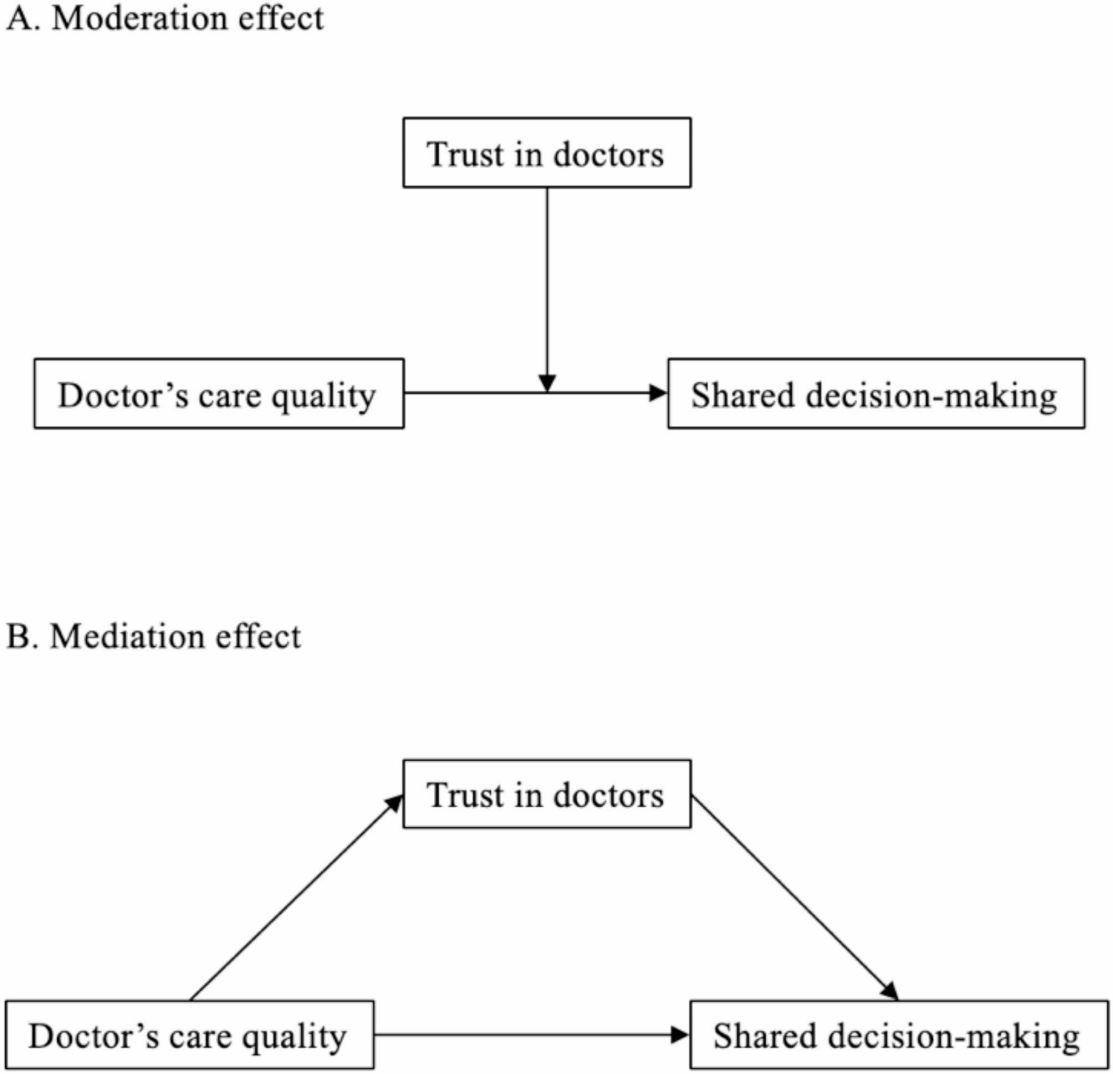
Conceptual framework

Concerning the mechanism underlying doctor’s care quality, patient’s trust in doctors, and engagement in SDM, trust in doctors may function as a moderator to buffer or exacerbate the effects of the quality of care provided by doctors on their engagement in SDM during medical consultations (see Fig. 1A). That is, higher levels of patient trust in doctors may lead to increased engagement in SDM by enhancing the positive impact of high-quality care provided by doctors [37]. Another potential mechanism is the mediation effects of trust in doctors. Namely, the level of doctor’s care quality influences patient’s level of trust in them, subsequently impacting patient engagement in SDM (see Fig. 1B). Studies have suggested that better quality of doctor’s care was associated with increased levels of trust in doctors [38]; and a higher level of trust in the doctor was linked to more involvement in SDM [34, 35, 39, 40]. However, there are limited empirical studies to explore whether trust in doctors mediated or moderated the relationship between doctor’s care quality and patient involvement in SDM among inpatients and outpatients. Understanding how trust influences the dynamics of these interactions can provide valuable insights into strategies for optimizing patient-centered care delivery and improving health outcomes.

To our knowledge, no previous study has comparing how doctor’s care quality and trust in doctors impacts SDM within inpatient and outpatient settings. Investigating this aspect could offer insights into the variations of SDM experiences between inpatient and outpatient settings, shedding light on the specific factors that shape patient involvement in decision-making across different healthcare scenarios. In light of these considerations, this study aimed to conduct a comparative study to explore the association between doctor’s care quality, trust in doctors, and shared decision-making among inpatients and outpatients in Hong Kong.

## Methods

### Data sources and study population

This study is based on data collected through a cross-sectional telephone survey of Specialist Outpatient Experience (Outpatients), which was conducted among 27 public specialist outpatient clinics between July 2021 and January 2022 in Hong Kong. Additionally, data from the Inpatient Experience (Inpatients) survey, conducted at 27 local public hospitals during the period from October 2019 to April 2020, was also included. The details of the questionnaires of Outpatients and Inpatients have been published elsewhere [41, 42]. All participants were invited to participate in the surveys through a random selection process.

### Study settings

In Hong Kong, both public inpatient and specialist outpatient services are integral parts of the healthcare system, offering comprehensive care tailored to the needs of different patient populations. In the inpatient setting, patients are typically assigned to regular doctors who oversee their care throughout their hospital stay. In contrast, patients attending outpatient clinics frequently do not have regular doctors, as they may see different specialists depending on availability and appointment schedules.

### Measurements

#### Doctor’s care quality and patient’s trust in doctors

Following the suggestion on the main domains of existing instruments assessing the doctor’s care quality [43], we selected three items to measure this variable: two items remained consistent in both the Outpatients and Inpatients survey questionnaires, aiming to assess patients’ evaluations of the doctor’s effectiveness (rated from 1 = “No” to 3 = “Yes, definitely”) in addressing their queries and the quality of care that the doctor provided (rated from 1 = “Very poor” to 5 = “Very good”); an analogous item in the two surveys to examine patients’ evaluation of the clarity of the doctor’s explanations regarding patient care and treatment (rated from 1 = “No” to 3 = “Yes, completely”). To maintain consistency in the Likert scale across all items, we condensed the original five-point Likert item measuring the quality of care received from the doctor into three broader categories (ranging from 1 = “Very poor” and “Poor”, 2 = “Fair”, and 3 = “Good” and “Very good”). Both the Outpatients (Cronbach’s alpha = 0.67) and Inpatients (Cronbach’s alpha = 0.83) surveys demonstrated acceptable internal reliability (Cronbach’s alpha > 0.6) for the three items [44]. The mean score was calculated by summing the responses across the three items and then dividing by three. Higher mean scores (ranging from 1 to 3) indicate a higher quality of doctor’s care. Furthermore, for the classification of respondents’ perception of their trust in the doctor’s examination and treatment, a single item with three responses (3 = “yes, definitely”, 2 = “yes, to some extent”, and 1 = “no”) was employed. The details of all the variables mentioned above and how they were measured are presented in Supplementary Table 1.

### Involvement in shared-decision making

Aligning with approaches in previous studies [45–47], patients’ perceived actual participation in decision-making was evaluated using a single item asking about their overall perception of involvement in decisions about their medical care and treatment. Responses were categorized into those who felt involved (“yes, definitely” and “yes, to some extent”) and those who did not (“no”). This approach accounts for varying levels of participation and better reflects real-world patient experiences, particularly in the Hong Kong context, where shared decision-making may not be widely understood [48]. Classifying only “yes, definitely” as involved while grouping “yes, to some extent” with “no” would set a higher threshold for involvement, potentially underestimating patient participation.

### Covariates

Participants’ sociodemographic information on age, gender, educational attainment, living status, and received allowance from the government were collected. Additionally, their self-reported health status and disability condition were also collected.

### Statistical analysis

First, we defined Outpatients and Inpatients as two subsets of respondents for the data analysis. We then conducted bivariate analyses using the Chi-square test or t-test to examine the differences in patients’ experience of SDM, sociodemographic and health characteristics, doctor’s care quality, and trust in doctors with statistical significance predefined at *p*-value < 0.05. Next, the main effect models (c path) of doctor’s care quality and involvement in SDM were conducted within each subset. The models were adjusted for the covariates mentioned above. Finally, the path analyses were conducted to explore whether trust in doctors mediated or moderated the association between doctor’s care quality and involvement in SDM. Regarding the moderating effects, the two-way interaction terms between doctor’s care quality and trust in doctors were added to the main effect models, and centered scores were used to calculate the interaction terms. In terms of the mediation analysis, the relationship between doctor’s care quality and trust in doctors (a path), the relation between trust in doctors and involvement in SDM (b path), and the relation between doctor’s care quality and involvement in SDM after controlling trust in doctors (c’ path) were examined with each subset.

## Results

### Characteristics of the study population

A total of 20,675 participants were included in the study (Table 1) after excluding all missing data. In the Outpatients survey, the final analytical sample consisted of 12,400 valid respondents (46.4% male, 53.5% female; 45.8% aged 60 years or younger, and 54.1% aged over 60). Few (28.1%) respondents possessed primary or no education, while the majority reported good health status (38.2% good, 57.2% fair), and did not receive government allowances (69%). A significant proportion of participants lived with their families (93.2%) and did not have disability conditions (92%). In the Inpatients subset, the sample consisted of 8,275 inpatients (52% men, 48% females; 59.3% aged 60 years or younger, and 40.7% aged over 60). Similarly, a small proportion (25.3%) of respondents possessed primary or no education (25.3%), had poor health status (7.8%), receive government allowances (24.8%), lived alone (7.4%), and had disability conditions (2.3%).

### Perceived involvement in decision-making

Overall, 7,973 outpatients and 3,972 inpatients perceived that they had been involved in decision-making about their treatment (see Table 1). In bivariate analysis among Outpatients, individuals who perceived that they did not participate in treatment decision-making were significantly more highly represented among the elderly aged over 60 (non-involved [60.8%] vs. involved [50.4%]), the lower education group (primary and below: non-involved [31.7%] vs. involved [26.1%]), patients with poor health (non-involved [5.9%] vs. involved [3.8%]), those with disability conditions (non-involved [10.3%] vs. involved [6.7%]), those living without families (non-involved [10.3%] vs. involved [4.8%]), and those receiving an allowance from the government (non-involved [35.3%] vs. involved [28.6%]). Similarly, among Inpatients, the percentages of respondents who perceived that they did not participate in treatment decision-making were significantly higher among females (non-involved [49.3%] vs. involved [46.7%]), the elderly aged over 60 (non-involved [47.9%] vs. involved [32.9%]), lower education group (primary and below: non-involved [31.4%] vs. involved [18.6%]), those living without families (non-involved [8.3%] vs. involved [6.4%]), and those receiving allowance from the government (non-involved [29.3%] vs. involved [19.9%]).

### Association between doctor’s care quality and perceived involvement in decision-making

The bivariate analysis (see Table 1) among the Outpatient subset indicated that the mean score of doctor-patient doctor’s care quality was significantly higher in the non-involved group (mean = 2.82, SD = 0.35), compared with the involved group (mean = 2.80, SD = 0.40). The results of the multivariable regression models (see Table 2) within this subset indicated that the likelihood of not being involved in treatment decision-making was significantly higher among the outpatients who perceived higher level of doctor’s care quality (OR = 0.91, 95%CI = 0.88–1.00, *p* = 0.001). Conversely, as shown in Table 1, among Inpatients, the non-involved group’s mean score of doctor’s care quality (mean = 2.78, SD = 0.35) was significantly lower than the involved group’s (mean = 2.81, SD = 0.35). After adjusting covariates (see Table 2), the risk of not participating in treatment decision-making was significantly higher among inpatients who perceived lower level of doctor’s care quality (OR = 1.29, 95%CI = 1.26–1.47, *p* < 0.001).

In the two subsets, patient involvement in decision-making was commonly associated with patients’ gender, age, education, health status, and living status. Specifically, older people (Outpatients: aged 41–60: OR = 0.85, 95%CI = 0.78–0.92, *p* < 0.001; aged > 60: OR = 0.68, 95%CI = 0.63–0.75, *p* < 0.001; Inpatients: aged 41–60: OR = 0.87, 95%CI = 0.80–0.94,*p* < 0.001; aged > 60: OR = 0.71, 95%CI = 0.64–0.77, *p* < 0.001), those with lower educational attainment (Outpatients: Secondary: OR = 1.08, 95%CI = 1.02–1.14, *p* = 0.01; Post-secondary: OR = 1.15, 95%CI = 1.07–1.24, *p* < 0.001; Inpatients: Secondary: OR = 1.26, 95%CI = 1.17–1.36, *p* < 0.001; Post-secondary: OR = 1.58, 95%CI = 1.44–1.73, *p* < 0.001), individuals with good health status (Outpatients: OR = 0.87, 95%CI = 0.77–0.97, *p* = 0.01; Inpatients: OR = 0.59, 95%CI = 0.53–0.66, *p* < 0.001), and individuals who live alone (Outpatients: OR = 0.64, 95%CI = 0.59–0.70, *p* < 0.001; Inpatients: OR = 0.89, 95%CI = 0.80–0.99, *p* = 0.03) were less likely to be involved in SDM.

### Moderation role of trust in doctors among inpatients

The path analysis (Table 3) indicated that trust in doctors significantly moderated the association between doctor’s care quality and engagement in decision-making among Inpatients (doctor’s care quality * Trust in doctors: β = 0.27, *p* < 0.001), while there no such moderation effects were evidenced among Outpatients (doctor’s care quality * Trust in doctors: β = 0.02, *p* = 0.26).

### Mediation role of trust in doctors among outpatients

**Fig. 2:**
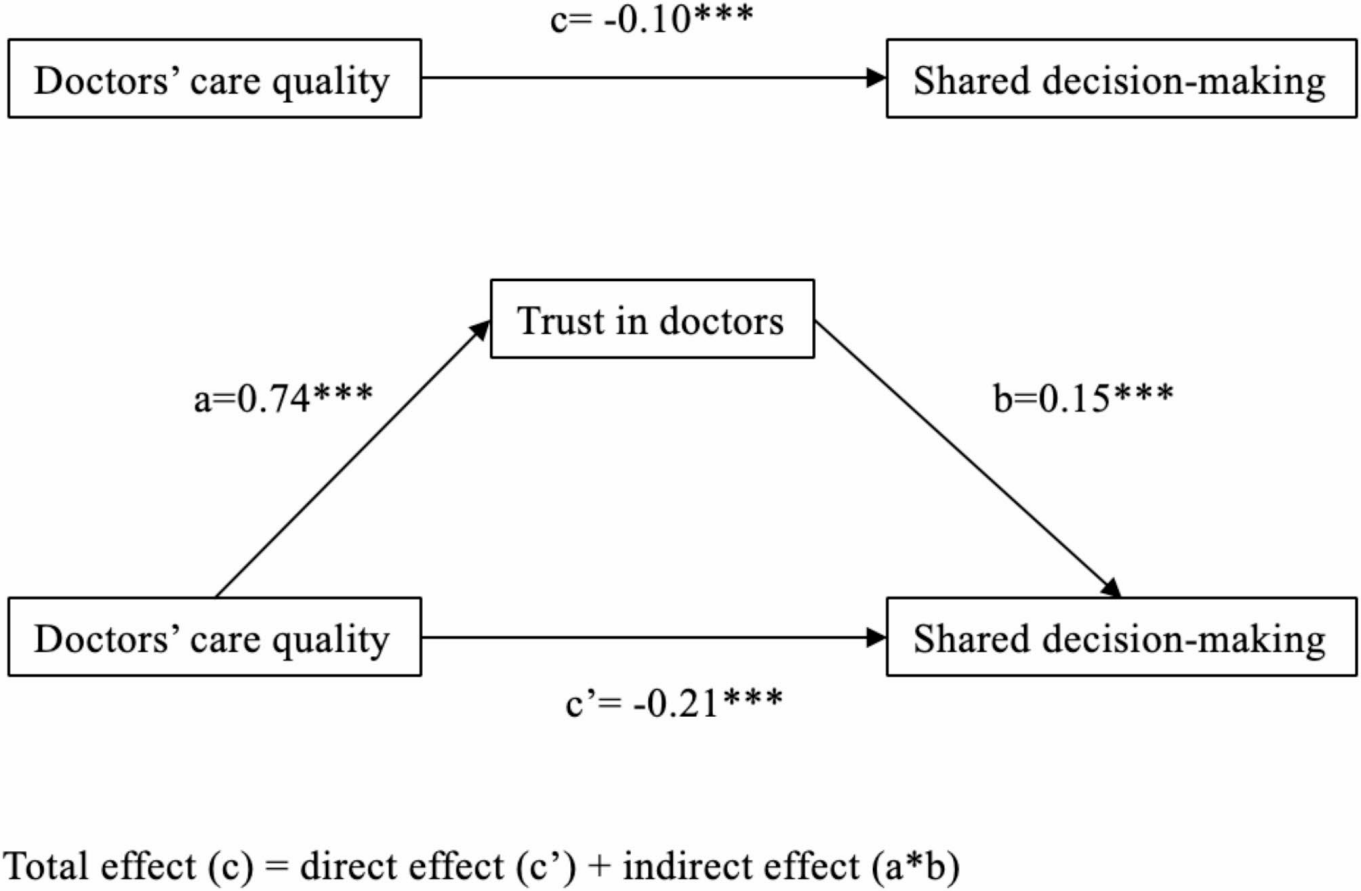
Suppressive mediation effect of trust in doctors among outpatients

As shown in Table 3 and Fig. 2, in the Outpatient dataset, we observed significant positive associations between doctor’s care quality and trust in doctors (a path: β = 0.74, *p* <.001), significant positive association between trust in doctors and involvement in SDM (b path: β = 0.15, *p* <.001). However, when looking at the overall relationship between doctor’s care quality and involvement in SDM, before considering trust in doctors (c path, total effect), as well as the direct effect of doctor’s care quality on involvement in SDM after accounting for trust in doctors (c’ path), both showed significant negative relationships. Notably, the direct effect (c’ path: β = −0.21, *p* < 0.001) was stronger than the total effect (c path: β = −0.10, *p* < 0.001), meaning trust in doctors played a suppressive role. In other words, trust in doctors counteracted part of the negative direct effect of doctor’s care quality on involvement in SDM, reducing its overall negative impact. Additionally, for inpatients, trust in doctors did not play a significant role in linking doctor’s care quality to involvement in SDM. This is because the effect of trust in doctors on involvement in SDM (b path: β = 0.002, *p* = 0.97) was not statistically significant.

**Table 1:** Characteristics of participants (*n* = 20,675)^#^

	Outpatients				Inpatients			
	Total (*N* = 12,400)	Non-involved (*N* = 4463)	Involved (*N* = 7937)	*P* value	Total (*N* = 8,275)	Non-involved (*N* = 4303)	Involved (*N* = 3972)	*P* value
Sex				0.87				0.02*
Male	5754 (46.4)	2066 (46.3)	3688 (46.5)		4298 (52.0)	2182 (50.7)	2116 (53.3)	
Female	6647 (53.6)	2397 (53.7)	4249 (53.5)		3997 (48.0)	2121 (49.3)	1856 (46.7)	
Age group				< 0.001 ***				< 0.001 ***
18–40	1452 (11.7)	410 (9.2)	1042 (13.1)		1581 (19.1)	632 (14.7)	949 (23.9)	
41–60	4236 (34.2)	1341 (30.0)	2895 (36.5)		3328 (40.2)	1610 (37.4)	1718 (43.3)	
> 60	6712 (54.1)	2712 (60.8)	4000 (50.4)		3366 (40.7)	2061 (47.9)	1305 (32.9)	
Education				< 0.001 ***				< 0.001 ***
Primary and below	3483 (28.1)	1413 (31.7)	2071(26.1)		2091 (25.3)	1352 (31.4)	739 (18.6)	
Secondary	5917 (47.7)	2081(46.6)	3836 (48.3)		4280 (51.7)	2188 (50.8)	2092 (52.7)	
Post-secondary	3000 (24.2)	969 (21.7)	2031 (25.6)		1904 (23.0)	763 (17.7)	1141 (28.7)	
Health status				< 0.001 ***				< 0.001 ***
Poor	563 (4.5)	263 (5.9)	300 (3.8)		645 (7.8)	271 (6.3)	374 (9.4)	
Fair	7095 (57.2)	2105 (47.2)	4990 (62.9)		4115 (49.7)	2154(50.1)	1961 (49.4)	
Good	4742 (38.2)	2095 (46.9)	2647 (33.3)		3515 (42.5)	1878 (43.6)	1637 (41.2)	
Disability condition^a^				< 0.001 ***				0.22
No	11,408 (92.0)	4003 (89.7)	7405 (93.3)		8083 (97.7)	4212 (97.9)	3871 (97.5)	
Yes	992 (8.0)	460 (10.3)	532 (6.7)		192 (2.3)	91 (2.1)	101 (2.5)	
Living status				< 0.001 ***				0.001 **
Live with families	11,559 (93.2)	4004(89.7)	7555 (95.2)		7661 (92.6)	3944 (91.7)	3717 (93.6)	
Live without families	841 (6.8)	459 (10.3)	382 (4.8)		614 (7.4)	359 (8.3)	255 (6.4)	
Received allowance from government				< 0.001 ***				< 0.001 ***
No	8556 (69.0)	2887 (64.9)	5669 (71.4)		6224 (75.2)	3041 (70.7)	3183 (80.1)	
Yes	3844 (31.0)	1576 (35.3)	2268 (28.6)		2051 (24.8)	1262 (29.3)	789 (19.9)	
Doctor’s care quality	2.81 (± 0.38)	2.82 (± 0.35)	2.80 (± 0.40)	< 0.001 ***	2.80 (± 0.36)	2.78 (± 0.37)	2.81 (± 0.35)	< 0.001 ***
Patient’s trust in doctors	2.83 (± 0.43)	2.82 (± 0.45)	2.83 (± 0.42)	0.26	2.87 (± 0.39)	2.86 (± 0.40)	2.88 (± 0.38)	0.07

*p*-values are obtained from the Chi-square test or t-test

*p < 0.05; **p < 0.01; ***p < 0.001

^#^All columns, except for the *p*-values column, are presented as the undefined number of participants (n) and column percentage (%). However, the columns for doctors' care quality and patients' trust in doctors are presented as the mean and standard deviation (SD)

^a^includes restriction in body movement, seeing difficulty, hearing difficulty, speech difficulty, mental illness/mood disorder, autism, specific learning difficulties, attention-deficit/hyperactivity disorder

**Table 2:** Association between doctor’s care quality and patient’s involvement in decision-making resulting from the main effects model(c path)

	Outpatients		Inpatients	
	OR (95% CI)	*P* value	OR (95% CI)	*P* value
**Intendent variable**				
Doctor’s care quality	0.91 (0.88–1.00)	0.001 **	1.29 (1.26–1.47)	< 0.001***
**Covariates**				
Sex: Female	0.95 (0.91–1.00)	0.04 *	0.92 (0.87–0.97)	0.007 **
Age group (Ref: 18–40):				
41–60	0.85 (0.78–0.92)	< 0.001***	0.87 (0.80–0.94)	< 0.001***
>60	0.68 (0.63–0.75)	< 0.001***	0.71 (0.64–0.77)	< 0.001***
Education (Ref: Primary and below):				
Secondary	1.08 (1.02–1.14)	0.01 *	1.26 (1.17–1.36)	< 0.001***
Post-secondary	1.15 (1.07–1.24)	< 0.001***	1.58 (1.44–1.73)	< 0.001***
Health status (Ref: Poor):				
Fair	1.44 (1.29–1.61)	< 0.001***	0.71 (0.64–0.79)	< 0.001***
Good	0.87 (0.77–0.97)	0.01 *	0.59 (0.53–0.66)	< 0.001***
Disability condition: Yes	0.78 (0.71–0.85)	< 0.001***	1.14 (0.95–1.37)	0.16
Received government allowance: Yes	0.95 (0.89–1.01)	0.09	0.88 (0.81–0.95)	0.001**
Live alone: Yes	0.64 (0.59–0.70)	< 0.001***	0.89 (0.80–0.99)	0.03*

*p < 0.05; **p < 0.01; ***p < 0.001

**Table 3:** Path analysis between doctor’s care quality, trust in doctors, and patient’s involvement in decision-making

	Outpatients		Inpatients	
	Coefficients	*P* value	Coefficients	*P* value
**Moderation effect**				
Doctor’s care quality * Trust in doctors	0.02	0.26	0.27	< 0.001***
**Mediation effect**				
Doctor’s care quality Involvement in decision-making (c path)	−0.10	< 0.001***	0.25	< 0.001***
Doctor’s care quality Trust in doctors (a path)	0.74	< 0.001***	0.71	< 0.001***
Trust in doctors Involvement in decision-making (b path)	0.15	< 0.001***	0.002	0.97
Doctor’s care quality + Trust in doctors Involvement in decision-making (c’ path)	−0.21	< 0.001***	0.25	< 0.001***

Adjusted for age, gender, undefined education, health status, disability condition, allowance, and living status

***p<0.001

## Discussion

### Main findings

Theoretically, a fundamental part of providing good doctor’s care quality is for doctors to convey information in a manner that patients can understand and enabling them to deliver services effectively [28, 29], leading to increased patient engagement in SDM. However, empirical evidence in this study painted a more complicated picture: inpatients who perceived a higher level of quality in doctor’s care with doctors were more likely to participate in treatment decisions, while outpatients who perceived higher doctor’s care quality were less likely to be involved in treatment decisions. These findings align closely with the principles of the NPT, which suggest that practices become normalized in daily routines based on how well they fit into the existing organizational contexts. To understand why different effects of doctor’s care quality on involvement in decision-making across the two contexts, we proposed to examine the NPT components of coherence (i.e., How participants make sense of the practice and understand its purpose and value) and cognitive participation (i.e., the engagement by participants).

Previous studies have reported the importance of having a regular doctor to help patients understand and be involved in SDM has been reported in previous studies [49, 50]. In Hong Kong, the inpatient setting typically offers continuity of care, with patients being attended to by a regular doctor or a consistent medical team throughout their hospital stay. This continuity fosters a stronger understanding of SDM, as the regular doctor can build rapport and continuously engage patients in their care decisions. Conversely, the outpatient setting is more fragmented, with patients often seeing different doctors during each visit [51, 52]. This lack of a regular doctor may hinder patients’ ability to fully understand and engage with SDM practices, as the consistency needed to build coherence and cognitive participation is disrupted. This difference in care continuity between inpatient and outpatient settings likely contributes to the varying impacts of doctor’s care quality on SDM involvement observed in this study. Additionally, while this study focused on the effectiveness of doctor’s care quality (i.e., whether doctors explained information clearly, provided clear answers, and delivered satisfactory care), the communication style — a factor not explored here — might impact patients’ cognitive participation in decision-making. In inpatient settings, where patients often have serious illnesses or need intensive care, healthcare providers may underscore the importance of a collaborative communication style [13, 53, 54]. This emphasis addresses the pressing urgency and intricate nature of inpatient care, thereby potentially facilitating increased patient engagement in SDM. Moreover, in inpatient settings, where patients often face serious health conditions or require intensive care, the high-stakes nature of decision-making may lead to greater patient involvement in SDM, particularly when they are under the care of a high-quality doctor. The hospitalization period also offers opportunities for patients to actively ask doctors questions if they have any queries, share their concerns, and engage in SDM [55]. Conversely, in outpatient settings, doctors may dominate the consultation [56]. Their communication styles with patients might be more directive or structuralized, focused on conveying information efficiently [57, 58], leading patients to feel less involved in decision-making. Considering that Hong Kong patients commonly prefer doctor-directed or paternalistic care and assume conventional passive roles in healthcare consultations [59–61], the directive communication style may significantly contribute to outpatients’ preference for relying on the doctors’ decision in short medical consultations. These results hold particular significance regarding barriers to shared decision-making, given that existing literature found many providers perceive themselves as already practicing shared decision-making while patients reported non-involvement in SDM [15, 62]. To address this issue, more health education programmes and workshops supporting patients to improve their health literacy and communication skills in healthcare consultation are needed to promote their involvement in medical decision-making.

This study also highlighted that building patients’ trust in doctors might be an important approach to consistently fostering SDM across various contexts. Specifically, the findings from the path analysis indicated two key insights. Firstly, the inpatient dataset suggested that trust in doctors significantly moderated the positive association between doctor’s care quality and involvement in SDM. In other words, inpatients’ level of trust in their doctors influenced how strongly doctor’s care quality was associated with patient engagement in SDM. Secondly, trust in doctors significantly suppressed the negative effect of doctor’s care quality on outpatients’ participation in decision-making. Despite the negative effect of doctor’s care quality on decision-making participation, trust in doctors mitigated this negative impact, suggesting that trust bolstered patients’ willingness to engage in decision-making despite potentially less favourable interactions. Overall, these findings underscore the importance of fostering trust in doctors as a means to enhance patient involvement in SDM.

In the present study, we observed that vulnerable groups, including females, the elderly, less-educated people, individuals with good health status, and those living alone were less likely involved in SDM. Theis finding is consistent with previous studies indicating that SDM might be more applicable to well-educated patients [1, 63, 64]. A higher level of education is usually linked to a higher level of health literacy, which enables patients to understand health information and then be actively involved in SDM. However, findings regarding the relationship between experiences of SDM and patient age and gender vary: a study of 233 patients in the US found no significant differences in SDM scores by age and gender [65]; one Irish study found that elderly patients and male patients had more positive experience regarding SDM [14]; while another study suggested that older age is significantly associated with more-passive perceptions of patient involvement in SDM [66]. The inclusion of a large sample size in the current study allowed for sufficient statistical power on the association between patient age, gender, and involvement in SDM. Additionally, this study found that patients with better health status are less likely to be involved in decision-making. One possible explanation for this is that individuals with good health status may have non-emergency admissions potentially reducing their dependence on shared decision-making processes [67, 68]. Concerning the association between patients’ living status (living alone vs. living with others) and involvement in SDM, it’s been shown that social support from families and significant others plays a significant role in facilitating SDM [34]. From these findings, we argue that clinicians should be aware that not all patients may be aware or ready to participate in SDM at a given moment. Therefore, it’s crucial to provide additional support and resources to vulnerable groups to ensure their active involvement in decision-making processes. These additional support could be instrumental in empowering vulnerable patients to actively participate in shared decision-making by improving their health literacy, enhancing communication with healthcare providers, involving family and social support, and ensuring they have the necessary resources and time to make informed healthcare choices.

### Implementation suggestions

The findings suggest that in inpatient settings, where continuity of care and high-stakes decision-making are prominent, patients are more likely to engage in SDM, particularly when cared for by high-quality doctors. In contrast, the fragmented nature of outpatient care and the more directive communication styles of doctors may hinder patient involvement. To address these challenges, it is crucial to foster a collaborative communication style, particularly in outpatient settings, to encourage patient participation [69, 70]. Moreover, trust in doctors plays a vital role in promoting SDM, as it strengthens the relationship between doctor’s care quality and patient involvement in decision-making. The study also identified vulnerable groups, including women, the elderly, less-educated individuals, and those in good health, as being less likely to engage in SDM. To overcome these barriers, it is essential to provide targeted interventions, such as health education programs, improved health literacy initiatives, and enhanced communication strategies that consider patients’ unique needs and circumstances [71, 72]. Clinicians should be aware that not all patients are ready to participate in SDM, especially in moments of vulnerability, and therefore, additional support, including social support, is needed to empower these patients. Ultimately, this study underscores the importance of a tailored approach to SDM that addresses the varying needs of patients across different settings, fostering an environment where all patients can be meaningfully involved in their healthcare decisions.

### Strengths and limitations

This is the first study to explore how doctor’s care quality and trust in doctors impact SDM within inpatient and outpatient settings. Although the study findings in this study are preliminary, they raise concerns that SDM in practice may be more complicated than what is intended by guidelines. The sample in the Outpatients and Inpatients subsets are representative, roughly aligning with the age and gender distribution of whole inpatients in 2019 [73] and specialist outpatients in Hong Kong in 2021 [74], respectively. Having a large and representative sample in this study enhanced statistical power to detect the relationships between doctor’s care quality and patient involvement in SDM in the data.

There are several limitations worth noting. Firstly, the nature of this cross-sectional study cannot establish a causal relationship between doctor’s care quality and patient involvement in SDM. Secondly, there might be recall bias among the participants. Thirdly, it is challenging to accurately measure patients’ involvement in SDM due to the complexity of this concept [75]. Even the most widely used measurement tools for SDM, such as the OPTION scale [76], predominantly emphasize physician behaviours and may overlook certain crucial aspects of engagement in decision-making interactions from the patients’ viewpoint. In this study, the measurement of patient involvement focused on the patients’ subjective overall feeling (rather than being) of involvement, consistent with methodologies observed in prior research [45–47]. Fourthly, measuring doctor’s care quality also presents challenges. As evidenced, there is no standard measurement tool for doctor’s care quality because of the high degree of heterogeneity in these measurements [77]. Although this study is based on secondary data and lacks a comprehensive scale to examine doctor’s care quality, we opted for items related to the primary domains of existing instruments used to assess these aspects. Fifthly, given that this study only includes quantitative data, further qualitative research is needed to explore how doctor’s care quality impacts patient involvement in decision-making, drawing on the principles of the NPT. Finally, data in this study were from the public hospitals and clinics in Hong Kong and may not be generalizable to other settings.

## Conclusion

This study suggests that inpatients may feel empowered to actively engage in decision-making when they perceive good doctor’s care quality with their doctors. Nevertheless, doctor’s care quality or patient-centeredness approaches did not ensure outpatients’ participation in decision-making processes. These findings underscore the importance of context-specific approaches to SDM implementation, recognizing the different challenges and dynamics in outpatient and inpatient settings. Establishing patient trust in doctors is a key approach to consistently fostering shared decision-making across various contexts. Furthermore, females, the elderly, individuals with lower educational attainment, those living alone, and those with good health status were found to be less likely involved in SDM. Therefore, it’s crucial to provide additional support and resources to these vulnerable groups to ensure their active involvement in decision-making processes.

## Supplementary Information

Supplementary Material 1.

## Data Availability

The data sets generated and/or analysed during the current study are not publicly available to protect the anonymity of participants but are available from the corresponding author on a reasonable request.
