# Supplementary material for: Implementation suggestions for shared decision-making: results from a comparative study of inpatients and outpatients experience surveys
Source: BMC Health Serv Res. 2025 Mar 11;25:362. doi: 10.1186/s12913-025-12507-0 (PMC11895247; doi:10.1186/s12913-025-12507-0)
Supplement: Supplementary file 1 — Supplementary Material 1. [file 12913_2025_12507_MOESM1_ESM.docx]

**Supplementary Table 1** Summary of measured variables in the two patient experience scale

|  | **Inpatients (2019)** | **Specialist Outpatients (2023)** |
| --- | --- | --- |
| Independent Variables |  |  |
| **Doctor-patient interaction** | q9. When you had important questions to ask a doctor, did your doctor provide a clear and understandable answer? | q24. When you had important questions to ask a doctor, did your doctor provide a clear and understandable answer? |
|  | q42. How would you rate the care you received from the doctors? | q52. How would you rate the care you received from the doctors? |
|  | q17. Were you told the detailed aspects of your condition, treatment, operation or procedure and its results in a way you could understand? | q23. Did the doctor explain the reasons for any treatment or action in a way that you could understand? |
| **Trust in doctors** | q10. Did you have confidence and trust in the doctors treating you? | q25. Did you have confidence and trust in the doctor examining and treating you? |
| Dependent Variable |  |  |
| **Perceived Involvement** | q24. Were you involved in decisions about your care and treatment? | q30. Were you involved in decisions about your care and treatment? |

Notes: This table outlines how the selected independent and dependent variables were measured and the wording used in the original scales of the two patient experience surveys.
